# Modeling of land subsidence using GIS-based artificial neural network in Yunlin County, Taiwan

**DOI:** 10.1038/s41598-023-31390-5

**Published:** 2023-03-11

**Authors:** Cheng-Yu Ku, Chih-Yu Liu

**Affiliations:** 1 Department of Harbor and River Engineering, National Taiwan Ocean University, Keelung, 20224 Taiwan; 2 Department of Civil Engineering, National Central University, Taoyuan, 320317 Taiwan

**Keywords:** Environmental sciences, Natural hazards

## Abstract

In this study, the land subsidence in Yunlin County, Taiwan, was modeled using an artificial neural network (ANN). Maps of the fine-grained soil percentage, average maximum drainage path length, agricultural land use percentage, electricity consumption of wells, and accumulated land subsidence depth were produced through geographic information system spatial analysis for 5607 cells in the study area. An ANN model based on a backpropagation neural network was developed to predict the accumulated land subsidence depth. A comparison of the model predictions with ground-truth leveling survey data indicated that the developed model had high accuracy. Moreover, the developed model was used to investigate the relationship of electricity consumption reduction with reductions in the total area of land with severe subsidence (> 4 cm per year); the relationship was approximately linear. In particular, the optimal results were obtained when decreasing the electricity consumption from 80 to 70% of the current value, with the area of severe land subsidence decreasing by 13.66%.

## Introduction

Land subsidence due to the overexploitation of water resources has been reported in numerous countries^1^. Moreover, anomalous recent global temperatures have affected environmental conditions and led to negative consequences, including the loss of water resource equilibrium and more frequent drought and flooding^2,3^. In the 1970s, researchers observed subsidence in the southern coastal areas of the Choshui delta on the west coast of central Taiwan^4,5^. The severity of this land subsidence increased, which resulted in damage to public infrastructure and numerous other problems. Therefore, mitigating land subsidence to prevent coastal hazards is critical to ensure that natural environmental resources can be developed sustainably^6,7^. The characteristics of the subsidence of Choshui delta are strongly affected by changes in the groundwater of inland and coastal areas. Over the past 10 years, subsidence has slowed in the coastal areas but has continued in inland areas^8^. Currently, of all areas of this delta, the central area exhibits the highest subsidence rate.

Various numerical, statistical, and artificial intelligence methods have been proposed for determining land subsidence risk^9,10^. Numerical approaches, such as the two-dimensional seepage method, quasi-three-dimensional seepage method, and three-dimensional fully-coupled method, are simple to implement and interpret. However, these methods may require long calculation times, and their input parameters might be difficult to determine^11,12^. Statistical approaches, such as time-series analysis, regression analysis, or Grey theory, are similarly easy to implement; however, they might have relatively low numerical accuracy^13–15^. Currently, artificial intelligence approaches, such as support vector machine (SVM) and artificial neural network (ANN), are frequently applied for land subsidence risk assessments. Although SVM can be effectively implemented in practice and requires few computational resources, it might not be applicable for large-scale engineering problems^16–18^. Increasingly scientifically accurate models are being developed in parallel with the growing understanding that these machine-learning approaches may be useful techniques for risk assessment. ANNs for land subsidence risk have been developed on the basis of remote sensing, geographic information system (GIS), and interferometric synthetic aperture radar data^19–22^. The computational process of an ANN algorithm is substantially more complicated than that of other methods. Moreover, an ANN algorithm requires setting numerous input parameters. Although it has a long computational time, its superior practical applicability offsets this cost^23–26^.

In this study, the land subsidence in Yunlin County, Taiwan, was modeled using an ANN. Two classes of variables affecting land subsidence were considered: those innate to the geology of the area and those related to human activity, which are denoted as “innate” and “human” variables, respectively. The human variables were groundwater withdrawal and agricultural land use, and the innate variables were percentage of fine-grained soil and average maximum drainage path length. The proposed GIS-based ANN model was employed to predict the land subsidence, and historical land subsidence data were used to evaluate the model predictions of land subsidence and establish the validity of the proposed model. The study methodology is described in the following text.

## Materials and methods

### Study area

**Figure 1:**
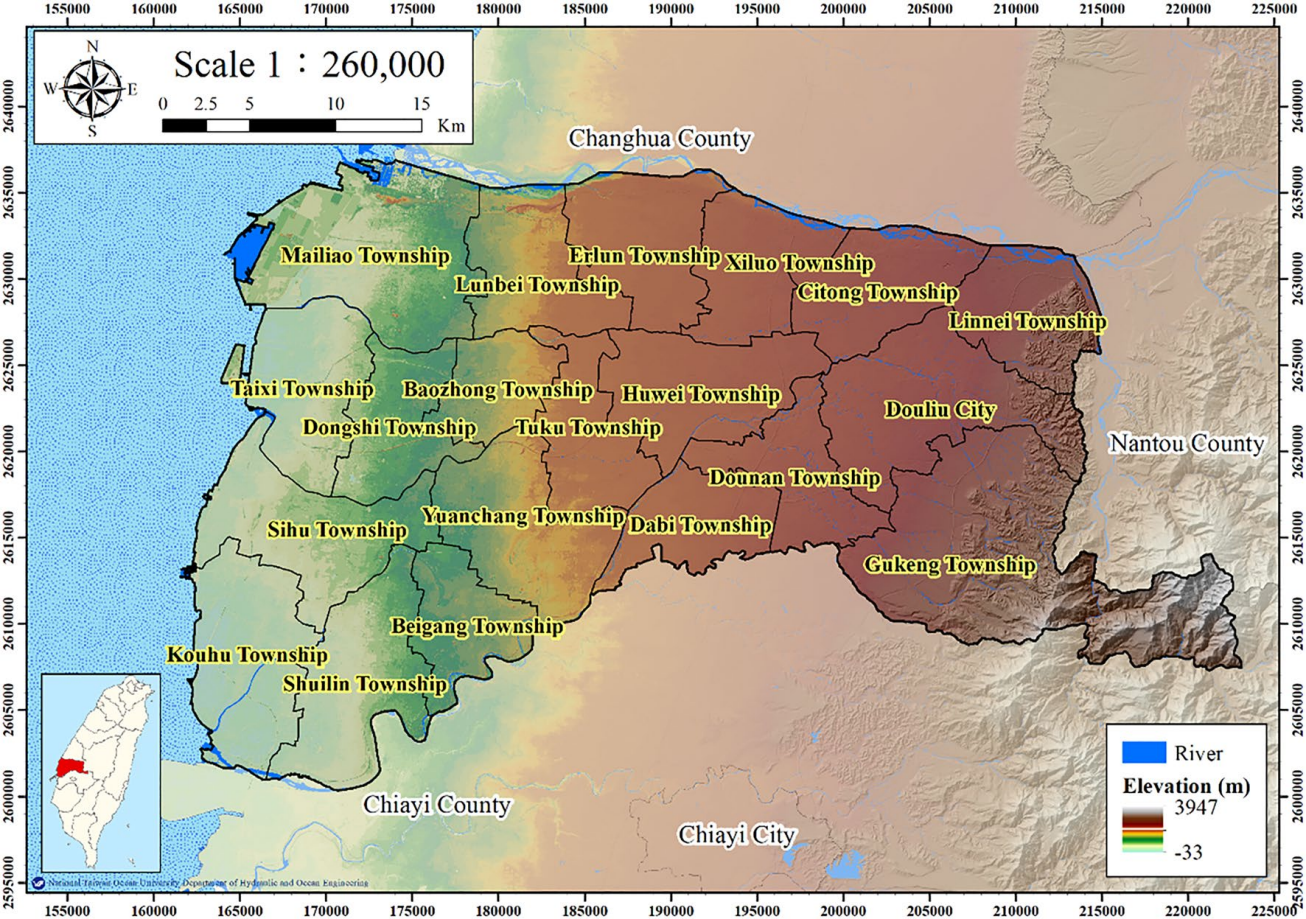
Topography of Yunlin County. This figure was created using ArcGIS 10.6.1 software.

Yunlin County is located in the south-central area of the Choshui delta in central Taiwan (Fig. 1). Yunlin County is flat; 10% of its area is hilly and 90% is covered by plains. This county is a key area for industry, agriculture, and aquaculture in central Taiwan. However, long-term overpumping has resulted in geohazards due to subsidence. According to the Water Resources Agency (WRA) of the Ministry of Economic Affairs of Taiwan, Yunlin County has a maximum annual subsidence rate of 5.5 cm—the highest in Taiwan in 2022. The areas with the greatest subsidence are in Tuku and Yuanchang Townships in the central Choshui delta.

### Geospatial data preparation

**Figure 2:**
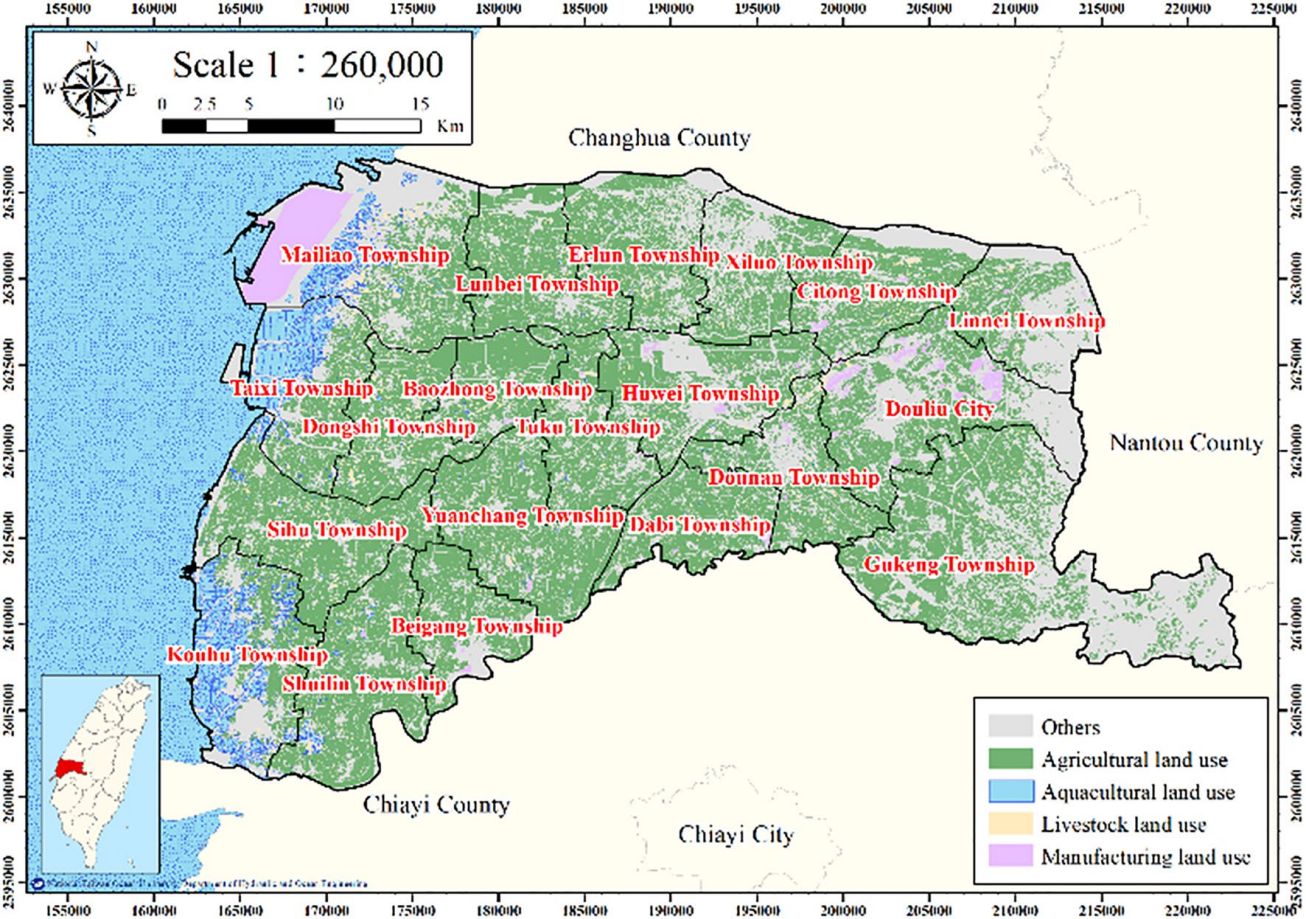
Land use in Yunlin County. This figure was created using ArcGIS 10.6.1 software.

The selected variables are listed in Table 1 (i.e., percentage of agricultural land use, electricity consumption of wells, percentage of fine-grained soil, average length of the maximum drainage path, and accumulated subsidence depth). Land use data with a resolution of 1 m were acquired from the National Land Surveying and Mapping Center (NLSC). Electricity consumption data with a resolution of 10 m were obtained from the Taiwan’s WRA. Borehole data were obtained from Taiwan’s Central Geological Survey (CGS) and WRA. The accumulated subsidence depth was calculated on the basis of data from WRA leveling surveys and multilayer compaction monitoring wells (MLCWs). Maps depicting these data were established using GIS spatial analysis to export geospatial layers (Fig. 2). The relationship between the innate and human factors causing land subsidence was evaluated using the ANN output.

**Table 1:** Source data.

No	Factors	Source data	Resolution (m)
1	Percentage of agricultural land use	Land use data Source: National Land Surveying and Mapping Center, Ministry of the Interior	1
2	Electricity consumption of wells	Electricity consumption per well for 91,607 wells Source: Water Resources Agency, Taiwan	250
3	Percentage of fine-grained soil	75 borehole logging data Source: Central Geological Survey and Water Resources Agency, Taiwan	250
4	Length of average maximum drainage path		250
5	Accumulated subsidence depth	Levelling surveys and 24 multilayer compaction monitoring wells Source: Water Resources Agency, Taiwan	250

#### Percentage of agricultural land use

The land use in Yunlin County is displayed in Fig. 2. Nine land use types were identified: agriculture, forestry, transportation, hydrological, construction, public, recreational, mining, and other. Most land in Yunlin County is agricultural land, and irrigation is widespread.

The percentage of agricultural land within 250 m of each MLCW was calculated using the ArcGIS buffer analysis tool to create a buffer polygon at a specified distance around input features to perform spatial analysis; the land areas were then calculated. The percentage of agricultural land use is defined as follows:

1 $$ L_{f} = \frac{{F_{a} }}{{T_{a} }}, $$

where $L_{f}$ denotes the percentage of agricultural land use, $F_{a}$ denotes the area of agricultural land in the considered land area, and $T_{a}$ denotes total considered land area.

#### Electricity consumption of wells

**Figure 3:**
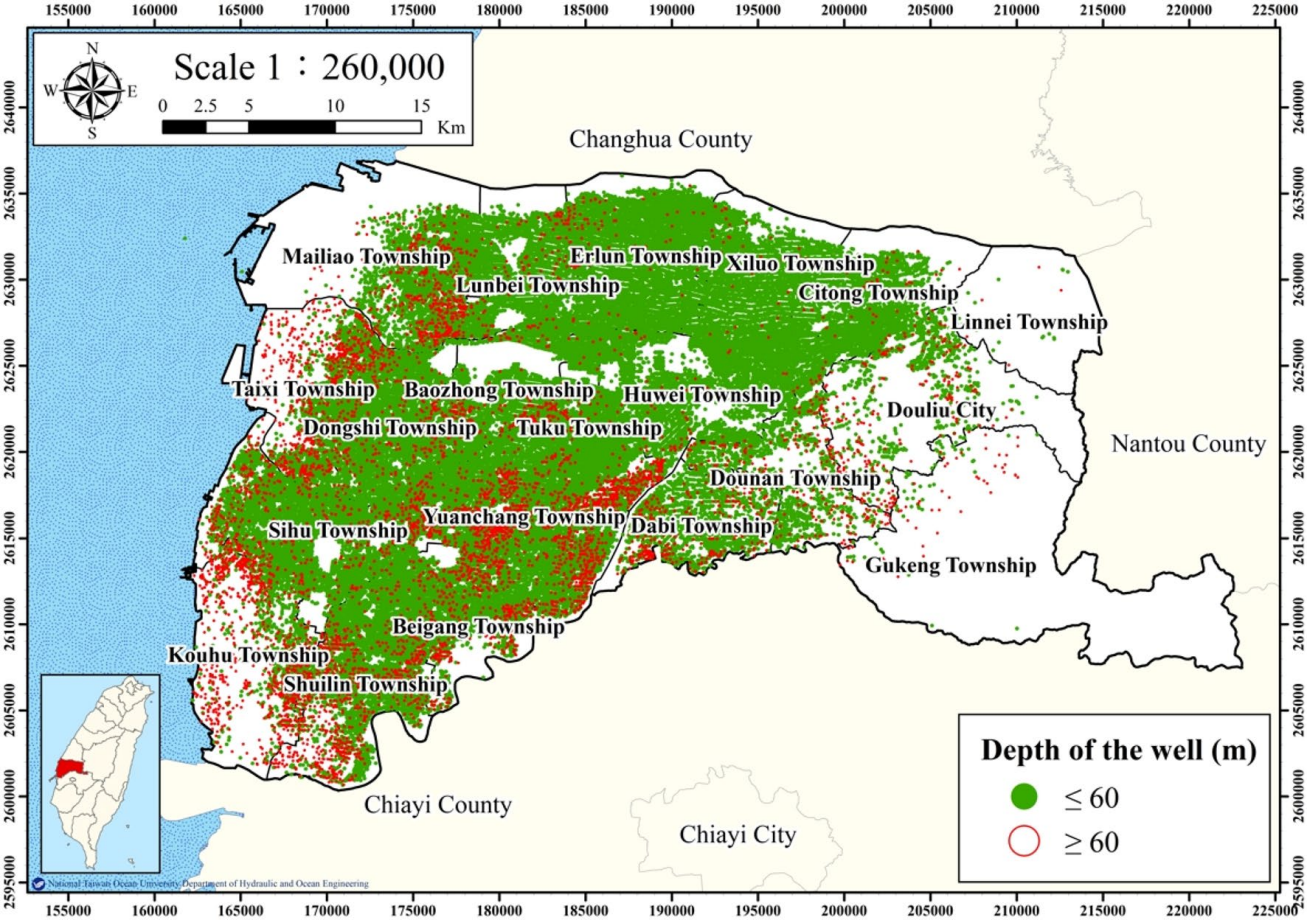
Distribution of managed wells in Yunlin County. This figure was created using ArcGIS 10.6.1 software.

Land subsidence is induced by the overexploitation of groundwater resources; thus, investigating groundwater usage is critical. Data for the groundwater usage related to well discharge are not available; however, electricity consumption by wells can be used as proxy indicator for groundwater usage. Figure 3 presents the distribution of managed wells in Yunlin County. Over 100,000 wells are located in the study area, and of these wells, 91,607 are irrigation wells, which were selected in this study. Most irrigation wells have a maximum depth of 60 m; thus, 84,349 irrigation wells with a depth of < 60 m were selected as a representative sample.

**Figure 4:**
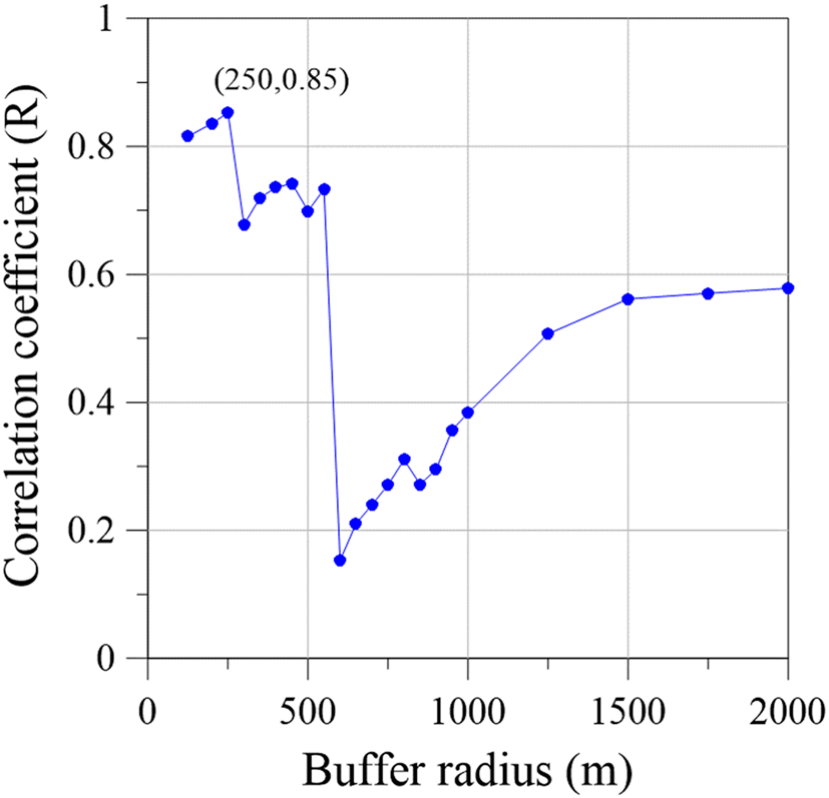
Plot of buffer radius versus correlation coefficient.

Land subsidence data were collected from 24 WRA MLCWs in Yunlin County with an average depth of 288.5 m. The land subsidence at an MLCW is attributable to all groundwater usage within its buffer radius. An appropriate buffer radius was determined by identifying the correlation coefficients between electricity consumption of wells and accumulated subsidence for various buffer radii between 150 and 2000 m and subsidence depths of 0–60 m (Fig. 4). The highest correlation coefficient of 0.85 was obtained for the buffer radius of 250 m; thus, 250 m was selected as the buffer radius for the subsequent analyses.

**Figure 5:**
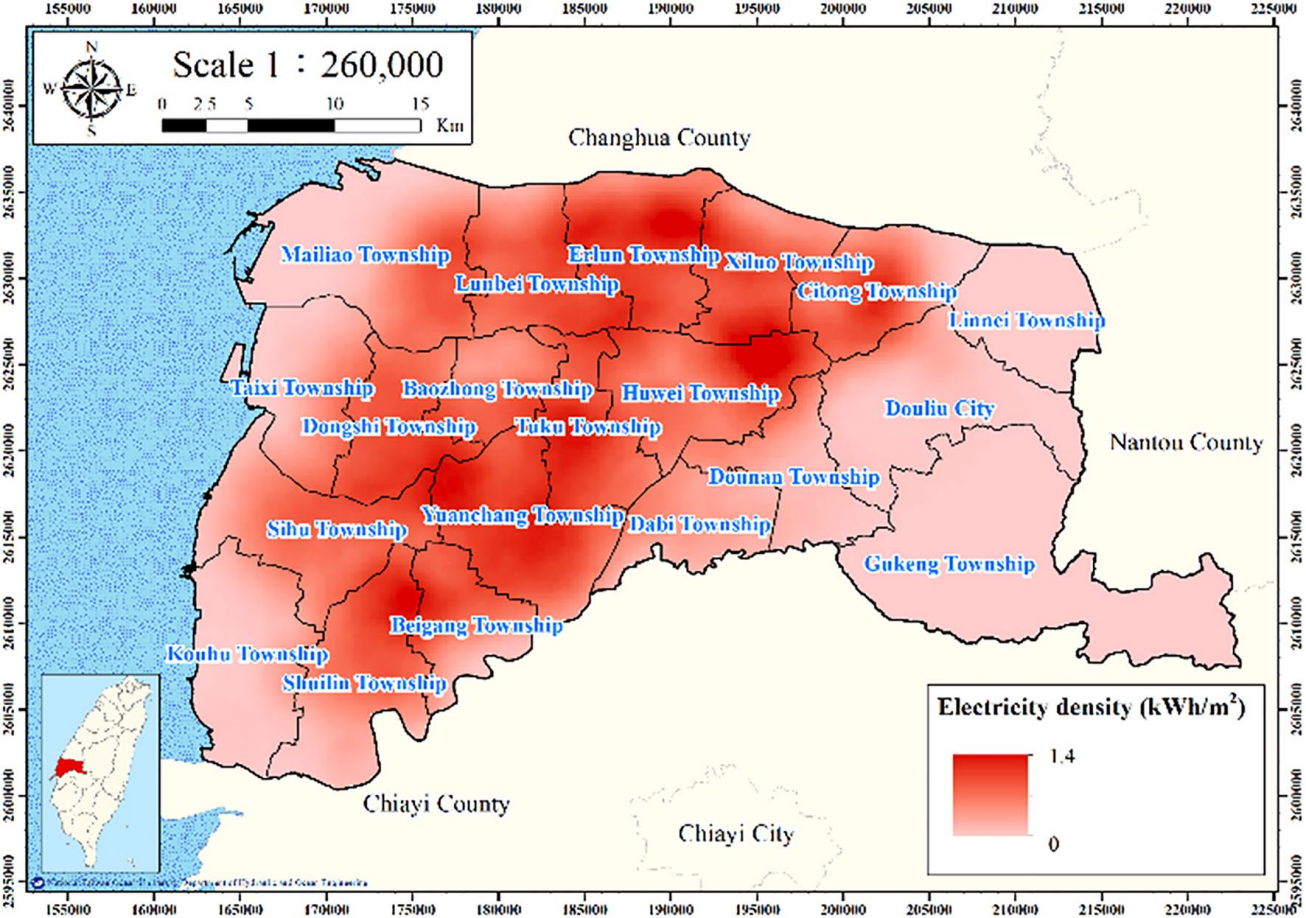
Electricity consumption of wells per unit square meter in Yunlin County. This figure was created using ArcGIS 10.6.1 software.

The electricity consumption data for 2015–2020 for the managed wells with a depth of 0–60 m within the buffer radius of each MLCW were collected. For spatially analyzing the electricity consumption of the managed wells of the area, the point density tool in ArcGIS was used to calculate the feature density in a neighborhood around the features. The electricity consumption of the managed water wells per unit area was calculated as follows:

2 $$ W_{e} = \frac{E}{{T_{a} }}, $$

where $W_{e}$ denotes the electricity consumption of the managed wells per unit area and *E* denotes the total electricity consumption of these wells in the considered land area. Figure 5 presents the electricity consumption of the managed wells of Yunlin County. This electricity consumption is clearly higher in the central part of Yunlin County than in its other parts.

#### Percentage of fine-grained soil

According to the unified soil classification system, fine-grained soils are whose ≥ 50% of content passes through the No. 200 sieve. Particles that pass through this sieve can typically not be observed with the naked eye, even with the aid of a magnifying glass. Fine-grained soils contain fine sand, silt, and clay. Percentage of fine-grained soil is defined as the ratio of the thickness of fine sand, silt, and clay layers in a borehole to the total drilling depth.

Percentage of fine-grained soil is calculated as follows:

3 $$ S_{f} = \frac{{H_{f} }}{{H_{t} }}, $$

where $S_{f}$ denotes the percentage of fine-grained soil, $H_{f}$ denotes the thickness of fine-grained soil, and $H_{t}$ denotes total drilling depth. In total, 75 borehole logs (24 from the WRA MLCWs and 51 from the CGS) were collected and used to calculate the fine-grained soil percentage.

**Figure 6:**
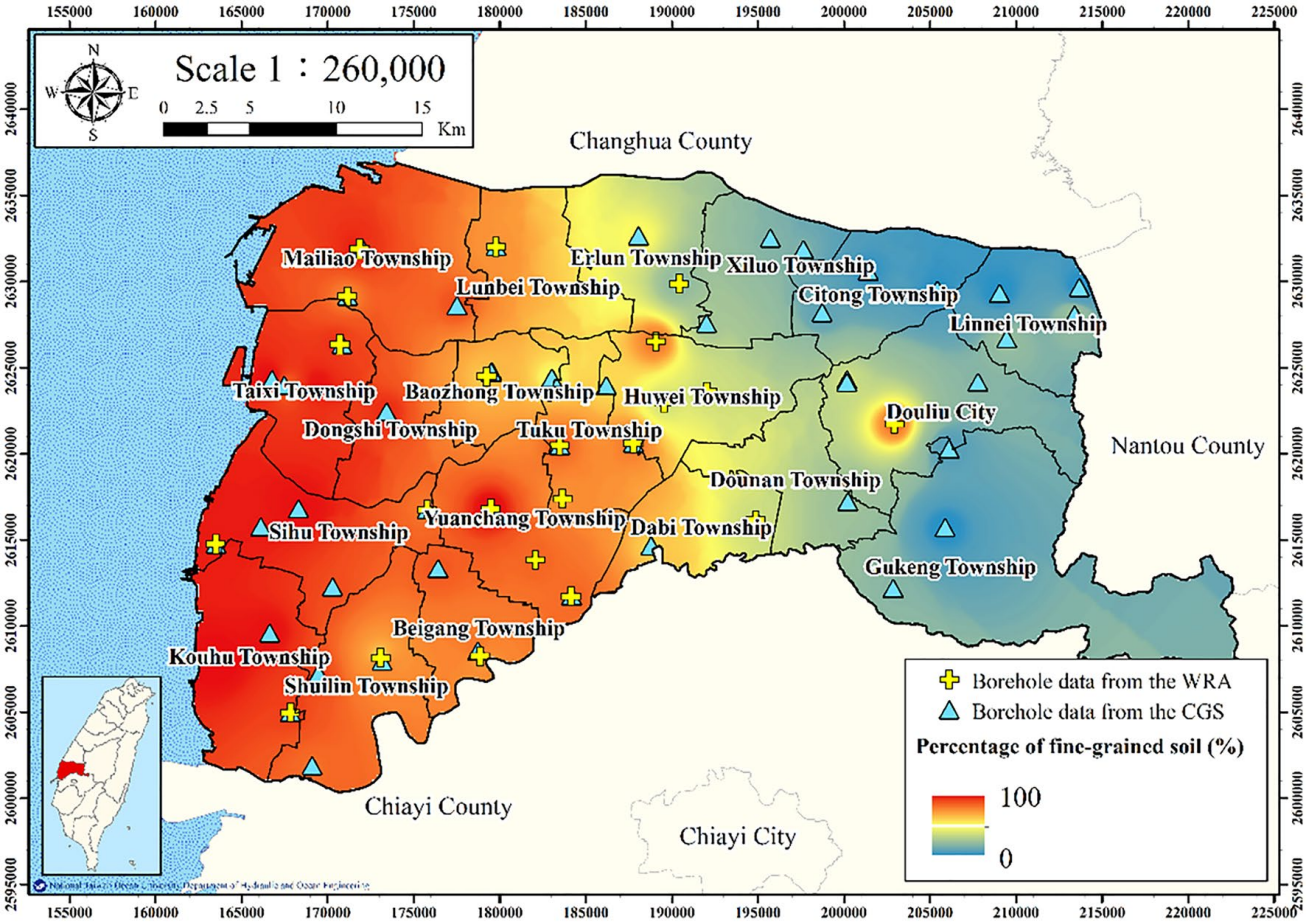
Percentage of fine-grained soil in Yunlin County. This figure was created using ArcGIS 10.6.1 software.

**Figure ****6** presents the WRA and CGS borehole locations and the corresponding fine-grained soil percentages. The percentage of fine-grained soil in the western coastal zone is clearly higher than that in eastern Yunlin County.

#### Average maximum drainage path length

**Figure 7:**
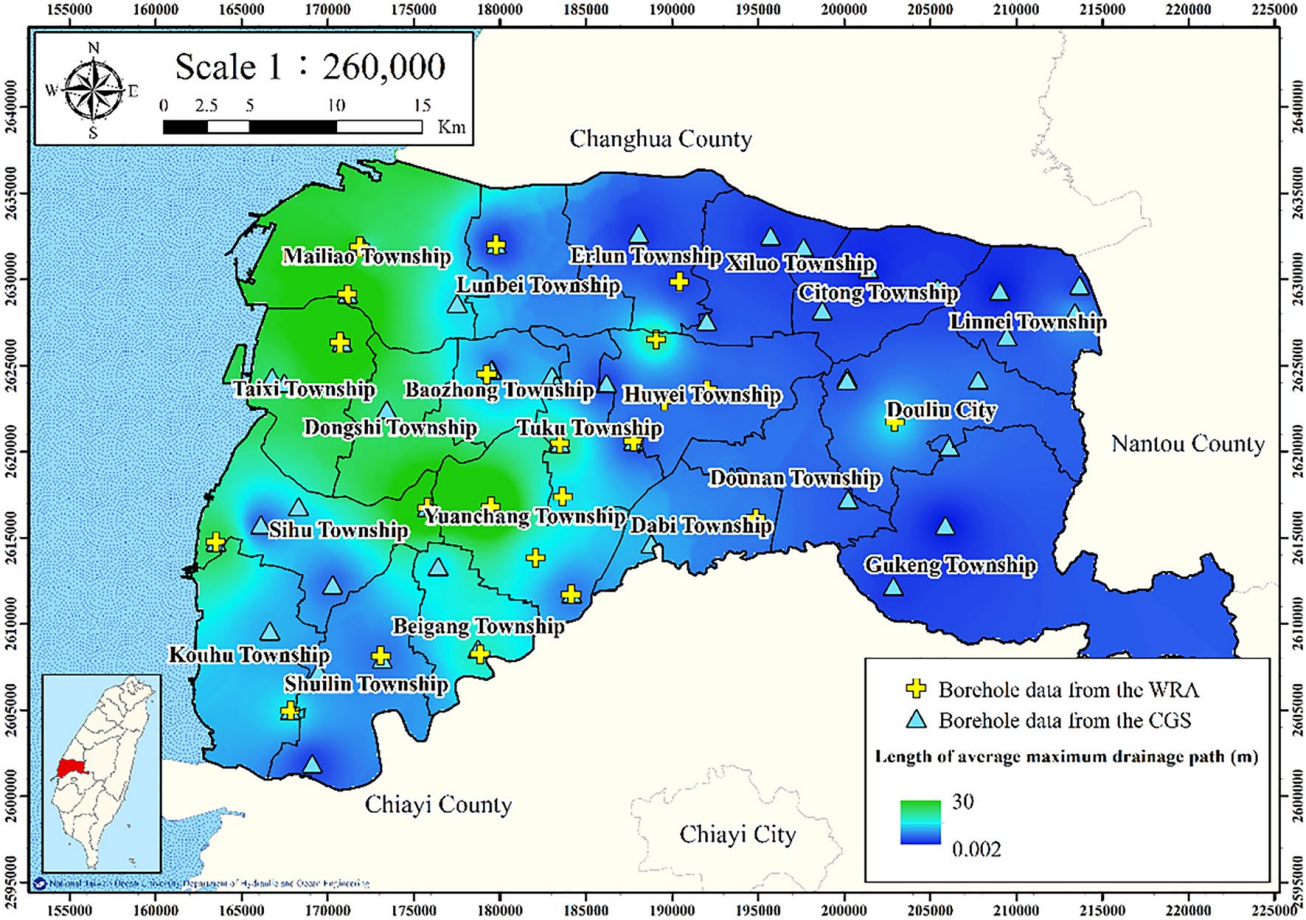
Average maximum drainage path length in Yunlin County. This figure was created using ArcGIS 10.6.1 software.

With consideration of the top and bottom drainage of the soil layer, average maximum drainage path length is defined as average drainage path length and is calculated as follows:

4 $$ H_{dr} = \frac{1}{n}\sum\limits_{i = 1}^{n} {(H_{if} /2)} , $$

where $H_{dr}$ denotes the length of the average maximum drainage path during compaction and $H_{if}$ denotes the thickness of fine-grained soil. If the stress of a saturated soil layer increases, the pore water pressure increases suddenly, which causes a reduction in the soil mass volume and subsequently results in settlement. The logging data from the 75 WRA and CGS boreholes were used to calculate the average maximum drainage path length (Fig. 7). The average maximum drainage path length in eastern Yunlin County is larger than that in western Yunlin County.

#### Accumulated subsidence depth

**Figure 8:**
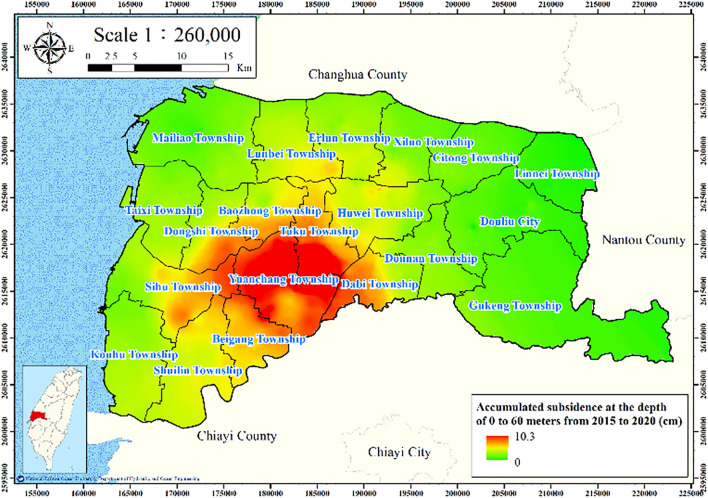
Accumulated subsidence from 2015 to 2020 at 0–60 m. This figure was created using ArcGIS 10.6.1 software.

The land subsidence from 2015 to 2020 in Yunlin County was evaluated using data from leveling surveys conducted at a depth of 0–60 m. The inverse distance weighting interpolation in GIS was applied to estimate subsidence. The results are presented in Fig. 8; the greatest subsidence is observed at Yuanchang and Tuku Townships.

### Artificial neural network

**Figure 9:**
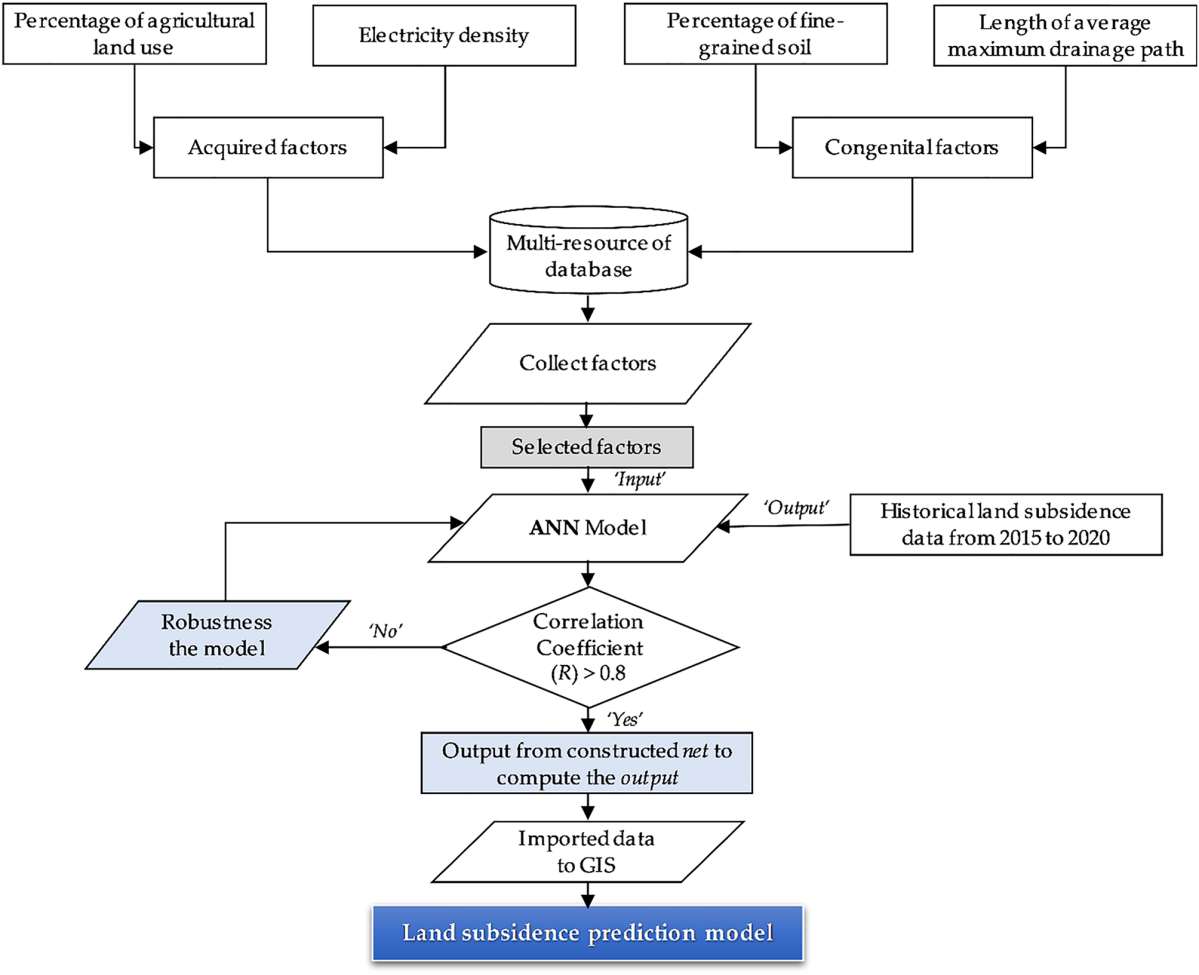
Flowchart of the proposed model.

Figure 9 displays a flowchart of the structure of the proposed ANN for modeling land subsidence in Yunlin County. The human variables were groundwater withdrawal and agricultural land use, and the innate variables were fine-grained soil percentage and average maximum drainage path length. A backpropagation neural network (BPNN) was used to evaluate land subsidence risk. The performance of the proposed GIS-based ANN prediction model was evaluated on the basis of the correlation coefficient (Fig. 9).

The functions of the training phase and the progress results are expressed as follows:

5 $$ y_{i} = F(X_{j} ) = \left[ {W_{oj} + \sum\limits_{i = 1}^{I} {\left( {W_{ij} x_{i} } \right)} } \right], $$

6 $$ Z_{k} = F(Y_{k} ) = \left[ {W_{ok} + \sum\limits_{j = 1}^{J} {\left( {W_{kj} y_{i} } \right)} } \right], $$

where $X_{j}$ and $Y_{k}$ denote the results obtained before adopting the activation function; $W_{oj}$ and $W_{ok}$ denote the bias weights for determining the threshold values; $F$ denotes an activation function ranging from 0 to 1; and $x_{i}$, $y_{i}$, and $Z_{k}$ denote the input, hidden, and output layers, respectively.

The hyperbolic tangent sigmoid function was selected as the activation function. The hidden and output layers are expressed as follows:

7 $$ y_{i} = F(X_{j} ) = F\left( {\frac{1}{{1 + e^{{ - X_{j} }} }}} \right), $$

8 $$ Z_{k} = F(Y_{k} ) = F\left( {\frac{1}{{1 + e^{{ - Y_{k} }} }}} \right), $$

Moreover, the error function is defined as follows:

9 $$ E = \frac{1}{2}\sum\limits_{k = 1}^{K} {\left( {\varepsilon_{k}^{2} } \right)} = \frac{1}{2}\sum\limits_{k = 1}^{K} {\left( {t_{k} - z_{k} } \right)^{2} } , $$

where* E* denotes the error, $t_{k}$ denotes the target value, and $\varepsilon_{k}$ denotes the error of each output node. Equation (9) was applied for error backpropagation weight training. The weighting between the hidden and output layers is expressed as follows:

10 $$ \Delta w_{jk} = \eta \times y_{i} \times \delta_{k} , $$

where $\eta$ denotes the learning rate. Equation (10) can be rewritten as follows:

11 $$ w_{jk} {(}n + {1)} = w_{jk} (n) + \Delta w_{jk} (n), $$

where *n* denotes the iteration number. Derived from the derivative with respect to $w_{ij}$, Eq. (9) is

12 $$ \frac{\partial E}{{\partial w_{ij} }} = \sum\limits_{k = 1}^{K} {\frac{\partial E}{{\partial z_{k} }}} \frac{\partial z}{{\partial Y_{k} }}\frac{{\partial_{k} }}{{\partial y_{i} }} \times \frac{{\partial y_{i} }}{{\partial X_{j} }} \times \frac{{\partial X_{j} }}{{\partial w_{ij} }} = - \Delta_{j} x_{i} , $$

13 $$ \Delta_{j} = F^{\prime}(X_{j} )\sum\limits_{k = 1}^{K} {\left( {\delta_{k} w_{jk} } \right)} . $$

The new weighting between the input and hidden layers is then given as follows:

14 $$ \Delta w_{ij} = \eta \times x_{i} \times \Delta_{j} , $$

15 $$ w_{ij} (n + 1) = w_{ij} (1) + \Delta w_{ij} (n). $$

**Figure 10:**
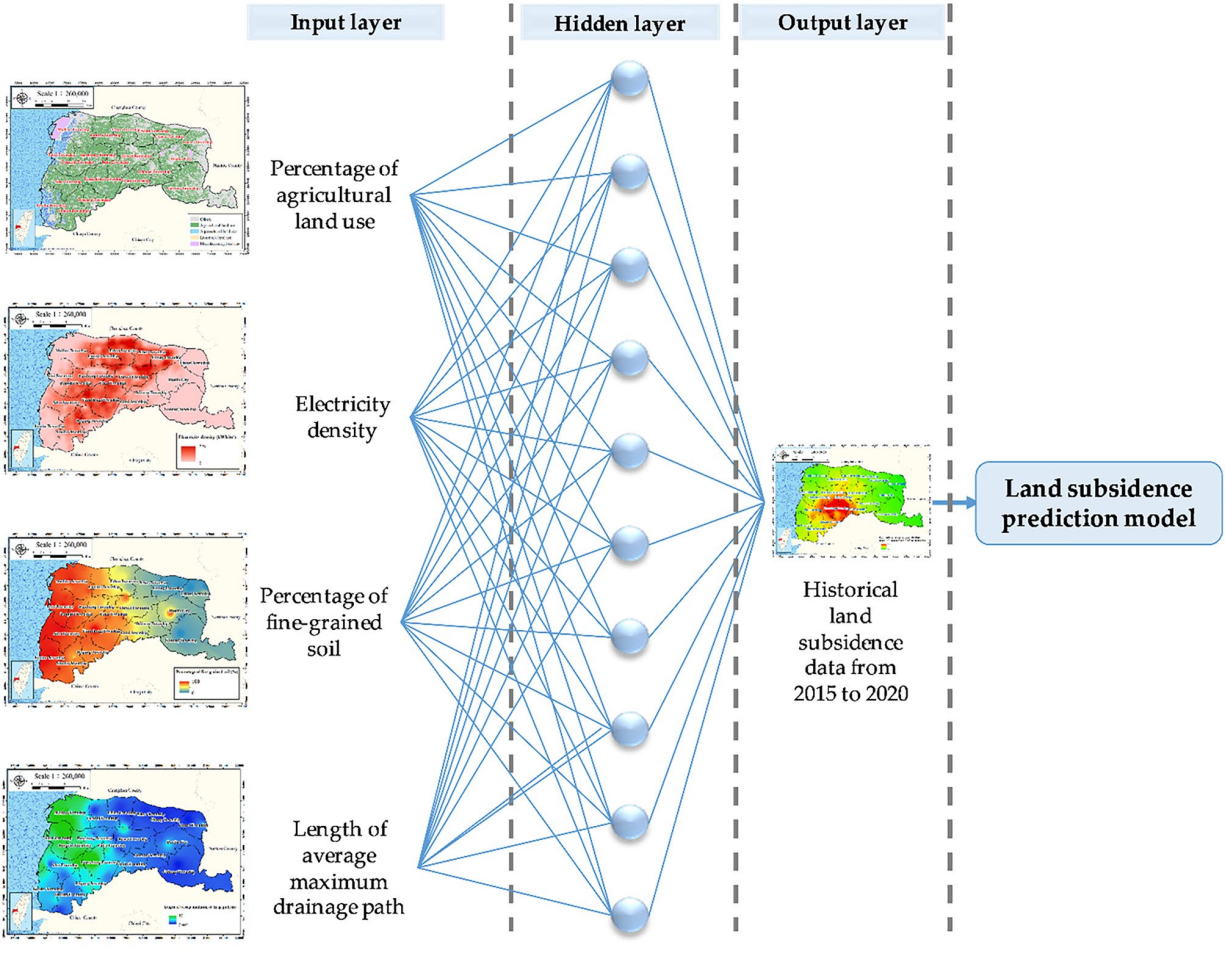
Proposed GIS-based ANN structure. This figure was created using ArcGIS 10.6.1 software.

The correlation coefficient was used as the performance metric for the proposed GIS-based ANN predictive model. This coefficient is defined as follows:

16 $$ R = \frac{{\sum\limits_{i = 1}^{n} {\left( {t_{i} - \overline{t}} \right)\left( {o_{i} - \overline{o}} \right)} }}{{\sqrt {\sum\limits_{i = 1}^{n} {\left( {t_{i} - \overline{t}} \right)^{2} \sum\limits_{i = 1}^{n} {\left( {o_{i} - \overline{o}} \right)^{2} } } } }}, $$

where* R* denotes the correlation coefficient, $t_{i}$ denotes the target value, $o_{i}$ denotes the output value, $\overline{t}$ denotes the average target value, and $\overline{o}$ denotes the average output value. Figure 10 illustrates the structure of the proposed model, which includes a BPNN algorithm, with four input variables: percentage of agricultural land use, electricity consumption of wells, percentage of fine-grained soil, and average maximum drainage path length.

The output was the predicted land subsidence in Yunlin County from 2015 to 2020. Thematic maps were established for percentage of fine-grained soil, length of average maximum drainage path, percentage of agricultural land use, electricity consumption of wells, and accumulated depth of land subsidence. A cell-based model was produced through GIS spatial analysis for 5607 cells in the study area, each of which had a size of 500 m. The buffer radius around the input features was 250 m; thus, in accordance with the maximum buffer zone length or diameter of 500 m.

**Figure 11:**
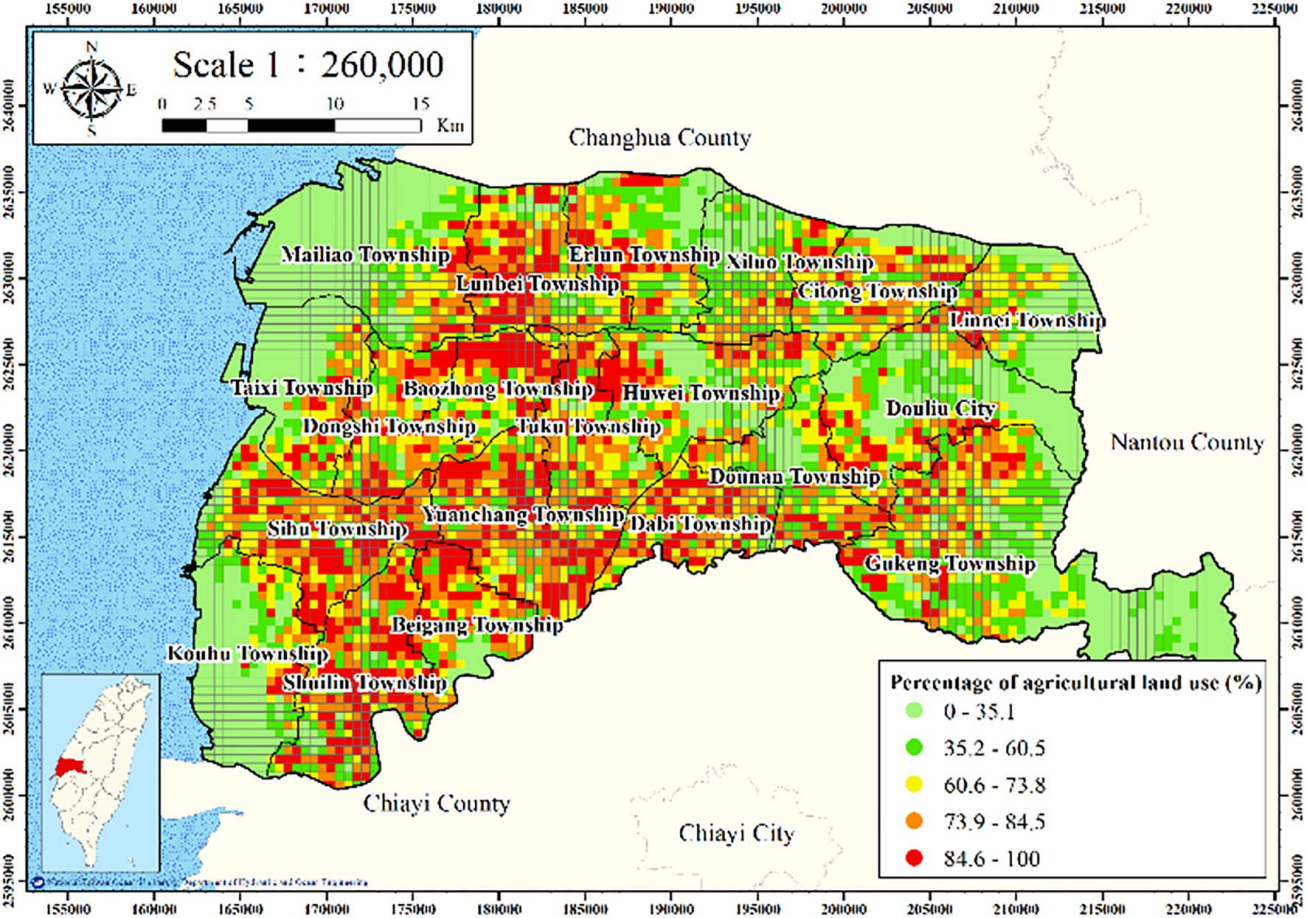
Cells of agricultural land use. This figure was created using ArcGIS 10.6.1 software.

**Figure 12:**
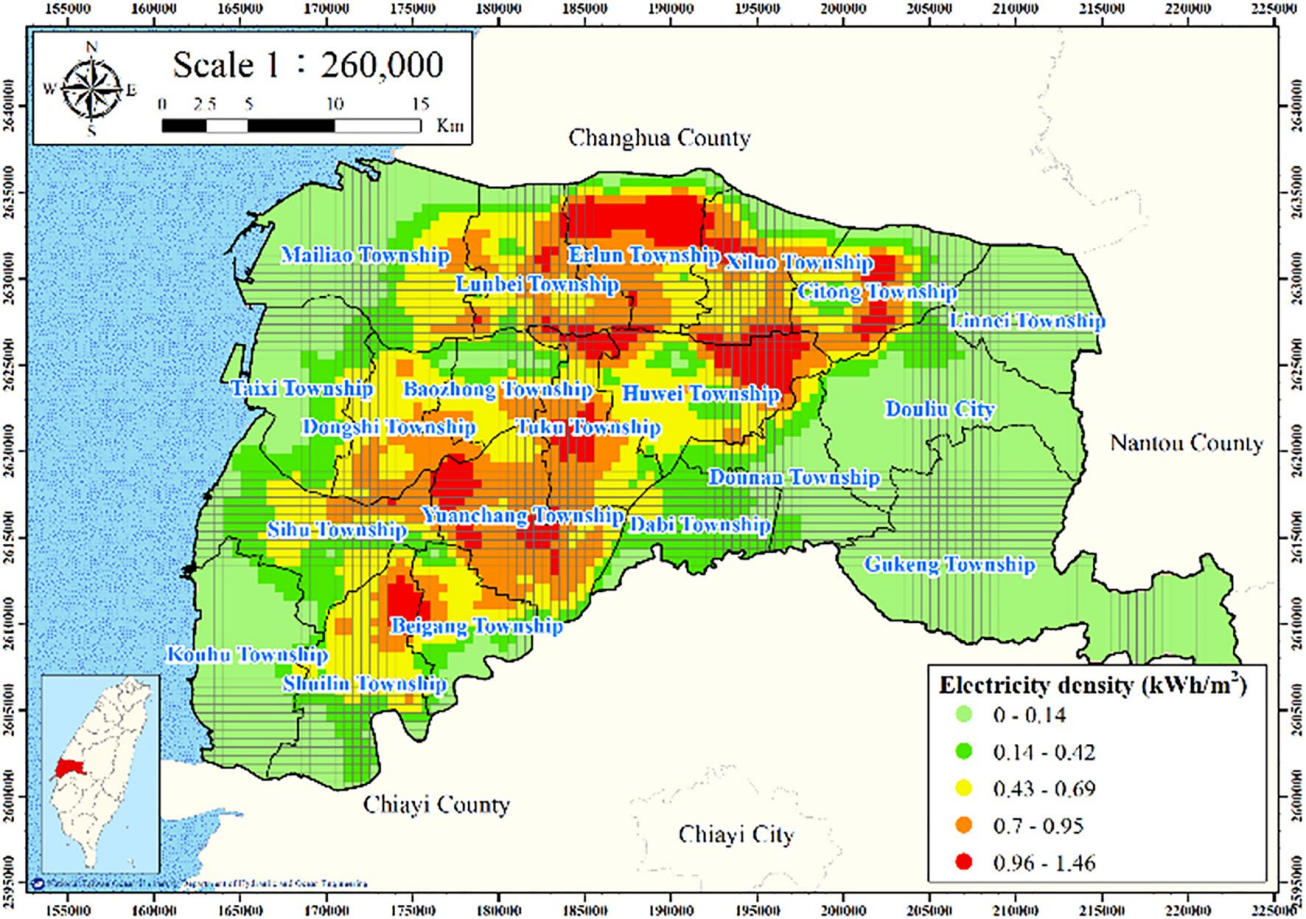
Cells of electricity consumption of wells per square meter. This figure was created using ArcGIS 10.6.1 software.

**Figure 13:**
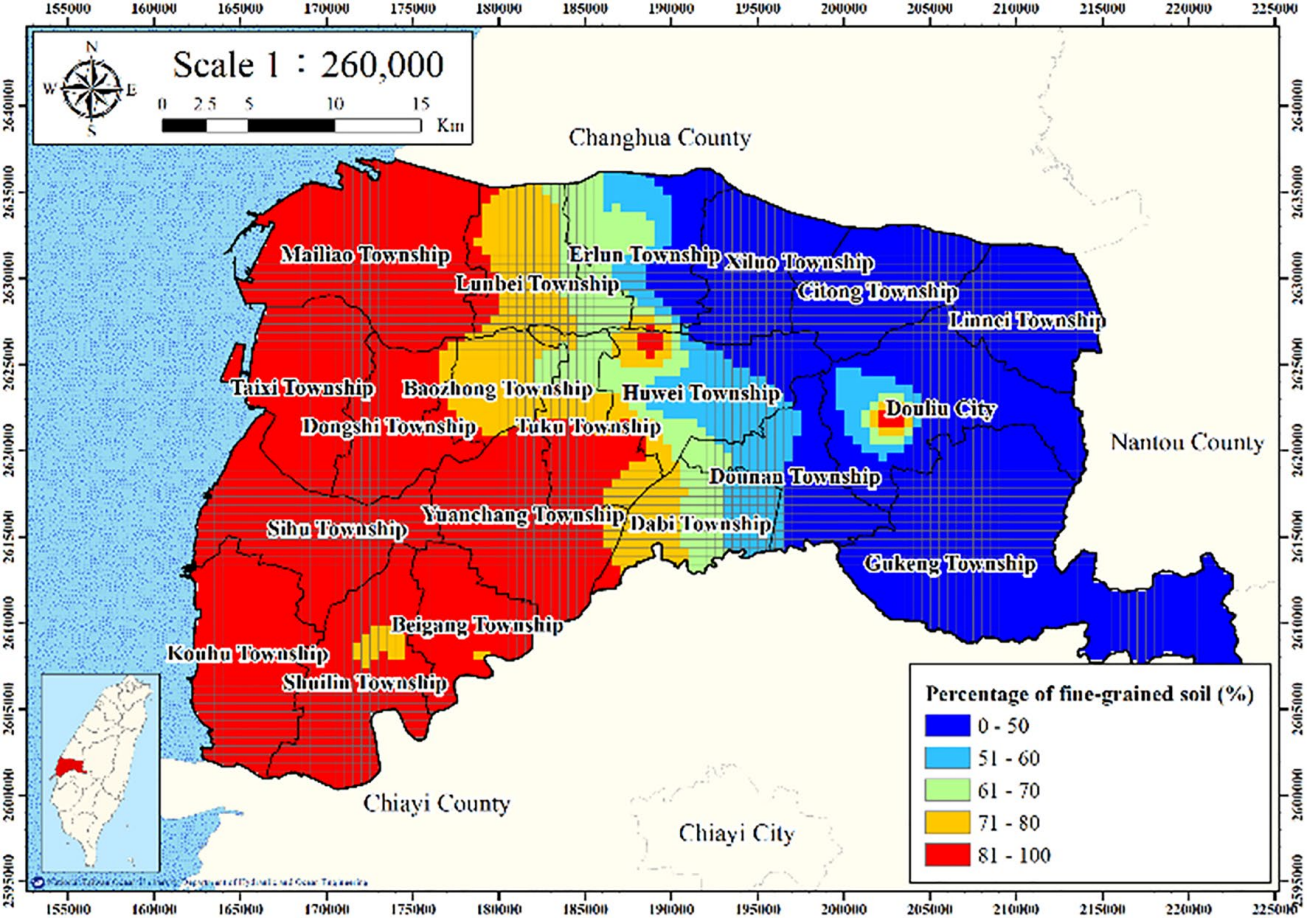
Cells of fine-grained soil. This figure was created using ArcGIS 10.6.1 software.

**Figure 14:**
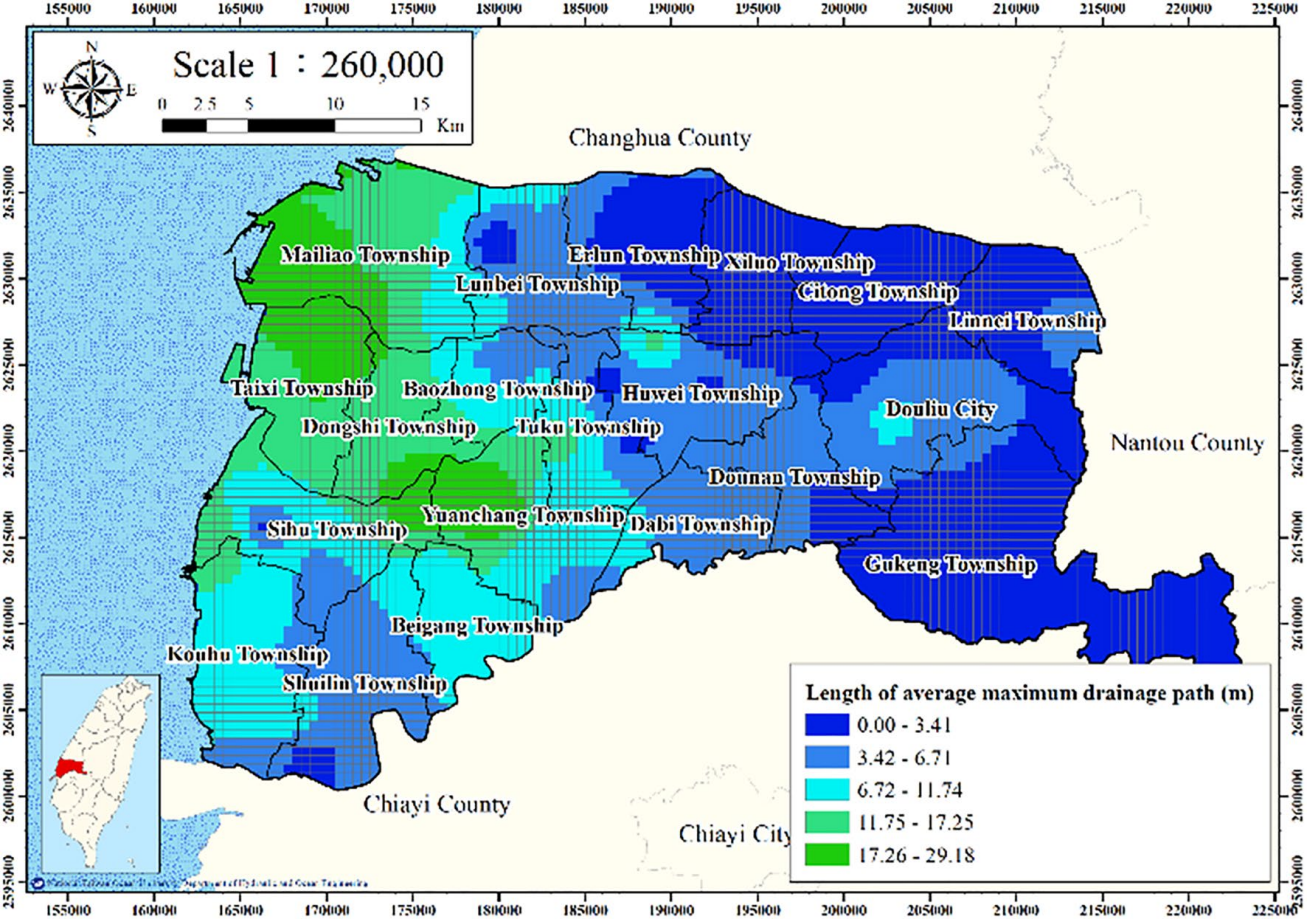
Cells of average maximum drainage path length. This figure was created using ArcGIS 10.6.1 software.

**Figure 15:**
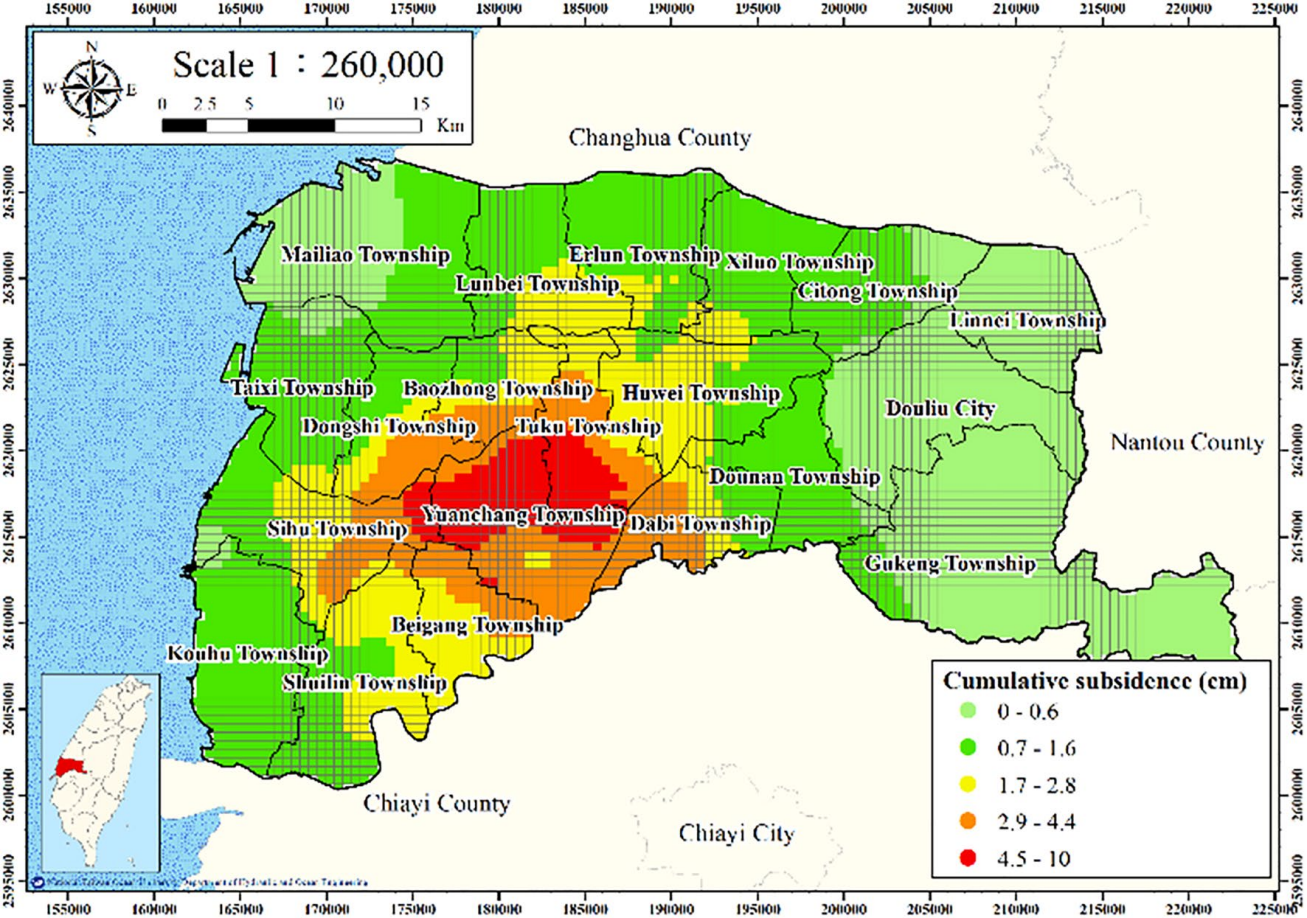
Cells of accumulated subsidence. This figure was created using ArcGIS 10.6.1 software.

Figures 11, 12, 13, 14 and 15 present the agricultural land use percentage, electricity consumption of wells per square meter, fine-grained soil percentage, average maximum drainage path length, and accumulated subsidence, respectively, at 0–60 m from 2015 to 2020 for the cell-based model. Figure 15 reveals that the highest subsidence is 10 cm. All the input data were normalized to the range of 0–1, and the initial weights were assigned randomly.

## Results

### Results obtained with the proposed GIS-based ANN model

The land subsidence data set was split into training, test, and verification data sets in the ratio of 70%:15%:15%. Table 2 lists the model parameters. The proposed land subsidence prediction model had four input layers, ten hidden layers, and one output layer. The Levenberg–Marquardt function was used for training, and the model performance was evaluated using the mean squared error (MSE).

**Table 2:** Model parameters.

	Initial value	Stopping criteria	Target value
Epoch	0	55	1000
Elapsed time	–	2 s	–
Performance	186	0.441	0
Gradient	312	0.721	10^−7^
mu	0.001	10^−5^	10^10^
Validation checks	0	6	6

Data division: random; training function: Levenberg–Marquardt function; performance function: MSE.

**Figure 16:**
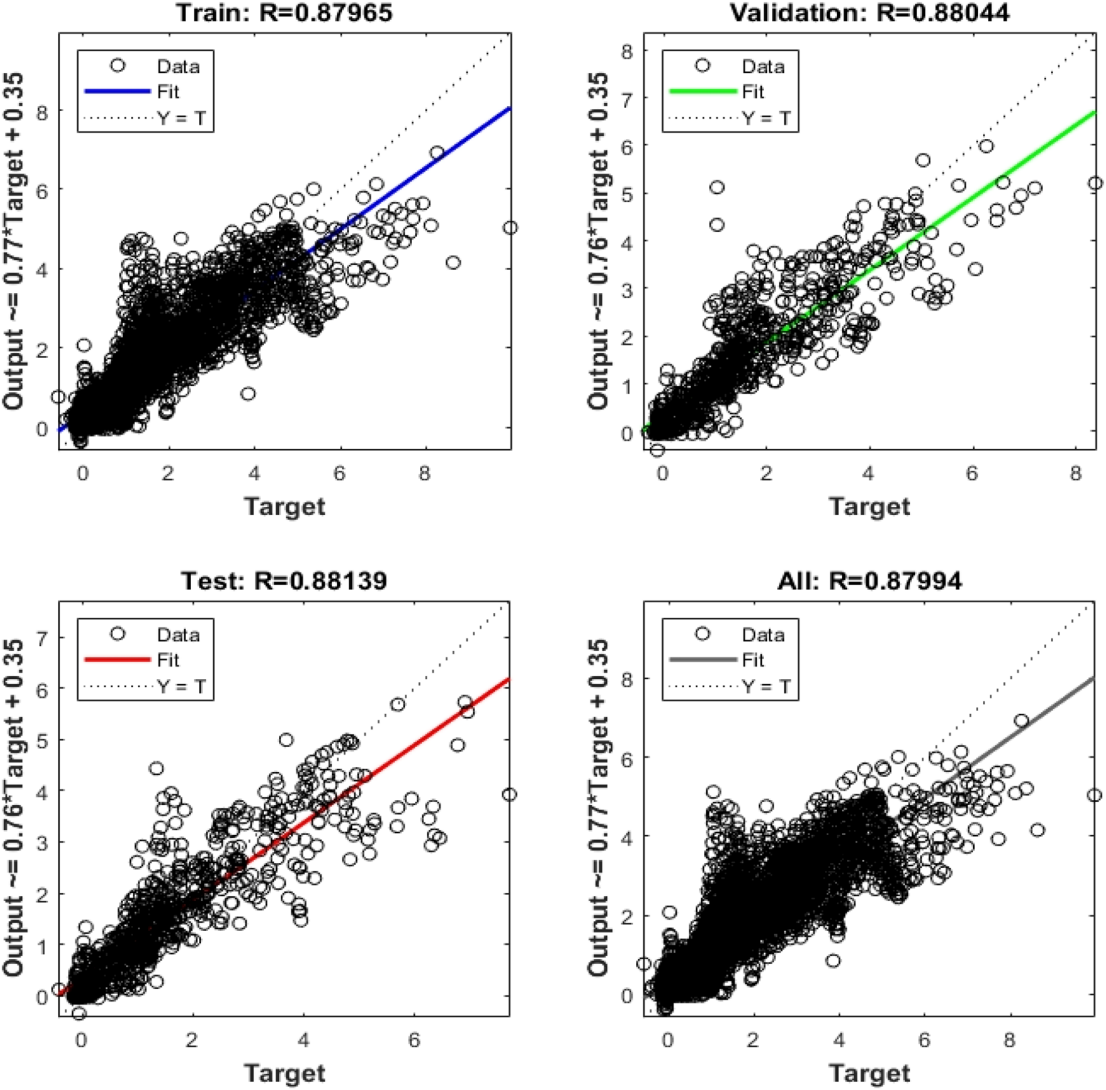
Correlation coefficients in training.

In training, the MSE was reduced from 10^2^ to 0.47 over 55 iterations. The MSE considerably decreased in the first five epochs before gradually declining until epoch 55. Figure 16 presents the training data for the correlation coefficient (*R*). The *R* values for the training, validation, and testing data sets were 0.880, 0.881, and 0.879, respectively, which indicated that the historical subsidence data and the predictions of the proposed model were strongly correlated. The aforementioned results validate that the proposed GIS-based ANN model can effectively predict land subsidence.

### Land subsidence prediction

#### Validation

To facilitate visualization and interpretation, the model outputs were exported using a GIS. The land subsidence was qualitatively analyzed using the natural breaks classification technique. The predicted subsidence values for areas with five risk classes—very high, high, moderate, low, and very low—were compared with the measured WRA subsidence values in these areas. Table 3 presents the accuracy of the proposed model. The model classified 20.98% (278.25 km^2^) of the area of Yunlin County as having high (16.7%) or very high (4.28%) risk of land subsidence. This area should be prioritized for land subsidence management.

**Table 3:** Land subsidence prediction accuracy.

Classification	Accumulated subsidence depth (cm)	Ths study		WRA (2021)		Relative error (%)
		Subsidence area (km^2^)	Percentage (%)	Subsidence area (km^2^)	Percentage (%)	
Very low	< 6	468.75	35.35	474.61	35.78	− 0.43%
Low	6 ~ 12	359.00	27.07	360.77	27.20	− 0.12%
Moderate	12 ~ 18	220.00	16.59	209.42	15.79	0.80%
High	18 ~ 24	221.50	16.70	213.69	16.11	0.59%
Very high	> 24	56.75	4.28	67.92	5.12	− 0.84%

**Figure 17:**
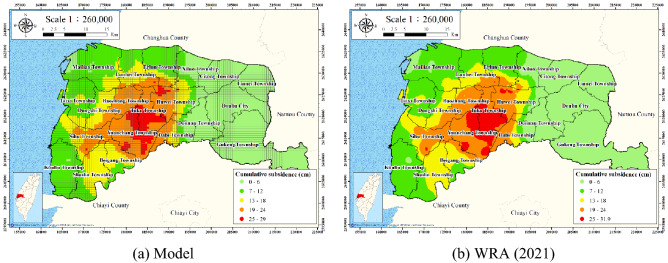
Land subsidence in Yunlin County. This figure was created using ArcGIS 10.6.1 software.

Figure 17 presents a comparison of the predicted and ground-truth (WRA) accumulated land subsidence for all strata in Yunlin County. Overall, the model predictions agree well with the WRA survey results.

The developed model was established for data from irrigation wells with a depth of at most 60 m. In conjunction with the WRA leveling survey data, the accumulated subsidence greater than 60 m can be directly obtained by subtracting the ground-level leveling survey data from the 0–60 m MLCW data. Accordingly, by superpositioning the predicted 0–60-m accumulated subsidence of the proposed model with the leveling survey data, the settlement of all strata can be obtained (Fig. 17).

#### Analysis of various electricity consumption scenarios

Practically, the pumping discharge cannot be measured for each of the over 100,000 wells in the survey area. However, electricity consumption is proportional to pumping discharge; thus, electricity consumption can be used as a proxy measurement for groundwater pumping discharge. Five scenarios of reducing electricity usage were considered: reducing electricity usage to 90%, 80%, 70%, 60%, and 50% of its original value. These scenarios are denoted Cases 1–5, respectively.

**Figure 18:**
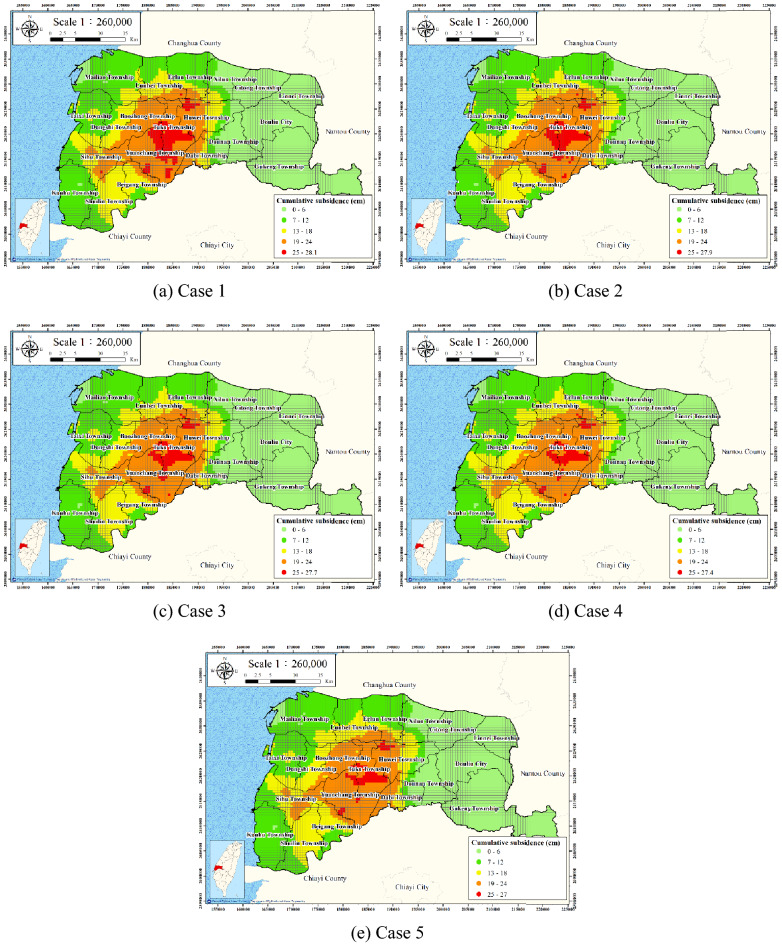
Land subsidence for each scenario. This figure was created using ArcGIS 10.6.1 software.

Figure 18 presents the prediction results for the five scenarios. According to the WRA, areas with an average annual subsidence rate of greater than 4 cm critically require subsidence management. Accordingly, the total subsidence area with an average annual subsidence rate of greater than 4 cm (critical subsidence area) was evaluated for each scenario (Table 4).

**Table 4:** Reduction of the critical subsidence area.

		Land subsidence area (km^2^)	Reducing subsidence area (km^2^)	Percentage of reducing subsidence area (%)	Cumulative percentage of reducing subsidence area (%)
Original electricity consumption		56.75	0	0%	0%
Reducing electricity consumption	Case 1: 90%	50.50	6.25	11.01%	11.01%
	Case 2: 80%	43.00	7.50	13.22%	24.23%
	Case 3: 70%	35.25	7.75	13.66%	37.89%
	Case 4: 60%	30.00	5.25	9.25%	47.14%
	Case 5: 50%	24.75	5.25	9.25%	56.39%

For Case 1 (90% electricity consumption), the critical subsidence area was 50.50 km^2^ (Fig. 18a), which represents a reduction of approximately 6.25 km^2^ (11.01% of the total area) compared with the 100% case. For Cases 2–5, the critical subsidence areas were 43.00, 35.25, 30.00, and 24.75 km^2^ (Fig. 18b–e), respectively. Table 4 reveals that the reduction of the critical subsidence areas for these cases was 7.50, 7.75, 5.25, and 5.25 km^2^, respectively, compared with that for the next-highest energy usage level.

**Figure 19:**
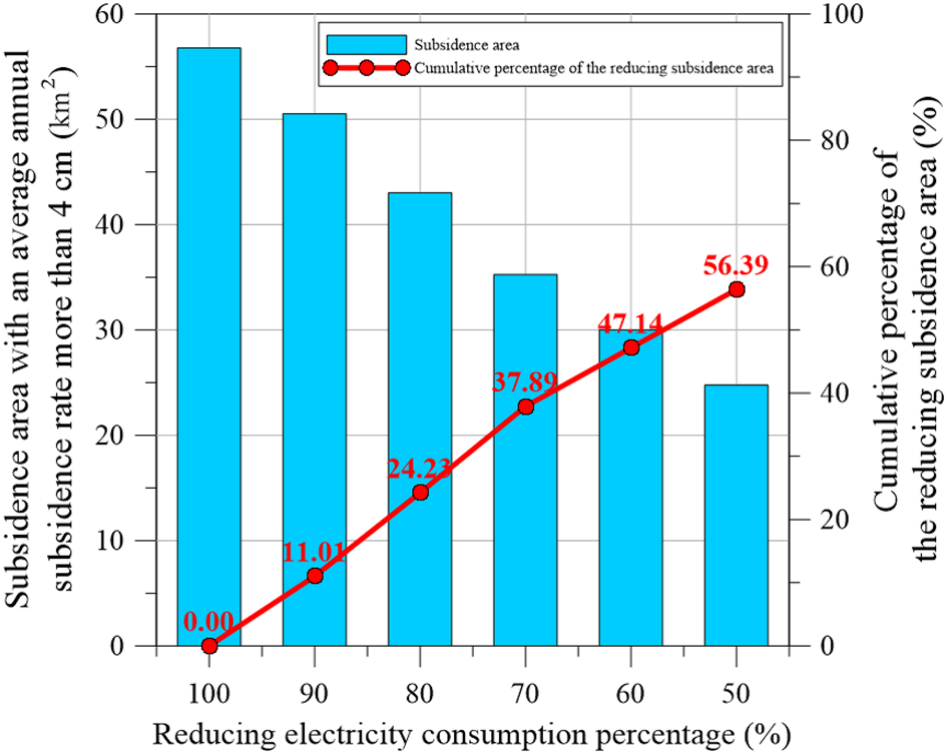
Relationship between reduction in electricity consumption and critical subsidence area.

Figure 19 reveals that the electricity usage reduction and the size of the critical area have an inversely proportional, approximately linear relationship. A decrease of approximately 10% in electricity consumption causes a decrease of 10% in critical subsidence area. Of the five scenarios considered in this study, reducing electricity consumption from 80 to 70% of the original consumption exhibited the best efficiency for decreasing the critical subsidence, with the decrease in the critical subsidence being 13.66% (Table 4).

The predictions of the proposed GIS-based ANN model are based on the assumption that a corresponding decrease occurs in the groundwater usage for irrigation. Moreover, an accumulated subsidence of greater than 60 m is not considered in the proposed model.

## Discussion

A GIS-based ANN model for predicting land subsidence in Yunlin County was developed in this study. The correlation coefficients for the training, validation, and testing data sets were 0.880, 0.881, and 0.879, respectively. Thus, the historical subsidence data and the model predictions were strongly correlated, which indicated that the proposed model effectively predicted land subsidence in the study area.

Moreover, the model was used to evaluate the effect of reducing electricity consumption to 90–50% of the current value on land subsidence in Yunlin County. The area with an average annual subsidence rate of greater than 4 cm was reduced by 7.50, 7.75, 5.25, and 5.25 km^2^ for the 80%, 70%, 60% and 50% scenarios, respectively. A reduction in the electricity consumption from 80 to 70% resulted in the maximum proportional reduction in the critical subsidence area (13.66%); thus, this scenario has the highest efficiency decreasing the critical subsidence. The findings of this research can be practically applied to develop sustainable management strategies for areas with severe subsidence.

## Conclusions

In this study, an ANN-based land subsidence prediction model was developed for Yunlin County, Taiwan. This pioneering study accurately predicted land subsidence in Yunlin County, Taiwan, and the conclusions of this study are as follows:

1. Maps of fine-grained soil percentage, average maximum drainage path length, agricultural land use percentage, electricity consumption of wells, and accumulated land subsidence depth were established for 5607 cells in Yunlin County through GIS spatial analysis. The proposed GIS-based ANN model, which included a BPNN, was then developed to predict the accumulated land subsidence depth. The predictions of the proposed model were compared with WRA leveling survey data to validate its accuracy.
2. The effect of reducing electricity consumption on land subsidence in Yunlin County was investigated, and reduction in electricity consumption has an approximately linear relationship with reduction in total area with severe land subsidence (> 4 cm per year).
3. A decrease of approximately 10% in the total electricity consumption caused a reduction of approximately 10% in the area with severe land subsidence. In particular, reducing the electricity consumption from 80 to 70% of the current value was the most efficient strategy, under which the severe land subsidence area decreased by 13.66%.

## Data Availability

The datasets of this study are available from the corresponding author on reasonable request.
